# A cross-sectional study observing the association of psychosocial stress and dietary intake with gut microbiota genera and alpha diversity among a young adult cohort of black and white women in Birmingham, Alabama

**DOI:** 10.21203/rs.3.rs-3146763/v1

**Published:** 2023-08-07

**Authors:** Rachel Knight, Yenni Cedillo, Suzanne Judd, Elizabeth Baker, Andrew Fruge, Douglas Moellering

**Affiliations:** University of Alabama at Birmingham; University of Alabama at Birmingham; University of Alabama at Birmingham; University of Alabama at Birmingham; Auburn University; University of Alabama at Birmingham

**Keywords:** psychosocial stress, diet quality, gut microbiota, young adults

## Abstract

**Background:**

The relationships between psychosocial stress and diet with gut microbiota composition and diversity deserve ongoing investigation. The primary aim of this study was to examine the associations of psychosocial stress measures and dietary variables with gut microbiota genera abundance and alpha diversity among young adult, black and white females. The secondary aim was to explore mediators of psychosocial stress and gut microbiota diversity and abundance.

**Methods:**

Data on 60 females who self-identified as African American (AA; n = 29) or European American (EA; n = 31) aged 21–45 years were included. Cortisol was measured in hair and saliva, and 16S analysis of stool samples were conducted. Discrimination experiences (recent and lifetime), perceived stress, and depression were evaluated based on validated instruments. Spearman correlations were performed to evaluate the influence of psychosocial stressors, cortisol measures, and dietary variables on gut microbiota genus abundance and alpha diversity measured by amplicon sequence variant(ASV) count. Mediation analyses assessed the mediating role of select dietary variables and cortisol measures on the associations between psychosocial stress, *Alistipes* and *Blautia* abundance, and ASV count.

**Results:**

AA females were found to have significantly lower ASV count and *Blautia* abundance. Results for the spearman correlations assessing the influence of psychosocial stress and dietary variables on gut microbiota abundance and ASV count were varied. Finally, diet nor cortisol was found to partially or fully mediate the associations between subjective stress measures, ASV count, and *Alistipes* and *Blautia* abundance.

**Conclusion:**

In this cross-sectional study, AA females had lower alpha diversity and *Blautia* abundance compared to EA females. Some psychosocial stressors and dietary variables were found to be correlated with ASV count and few gut microbiota genera. Larger scale studies are needed to explore the relationships among psychosocial stress, diet and the gut microbiome.

## Introduction

For decades, research has shown how stress gets “under the skin” by activating neuroendocrine, cardiovascular, and metabolic systems,^1–3^ ultimately contributing to the development of diseases and conditions such as hypertension, atherosclerosis, diabetes mellitus, nonalcoholic fatty liver disease, Alzheimer’s, depression, and cancer. Stress, defined as a state of threatened homeostasis,^4^ is becoming more pervasive through psychosocial, environmental, and cultural means in the United States,^5^ with discrimination recognized as a stressor likely contributing to ongoing health disparities^6^. Reported experience of discrimination has been associated with cardiometabolic risk factors including elevated blood pressure and pulse,^7,8^ stress hormones,^9^ inflammatory cytokines,^10,11^ obesity,^12,13^ and mental health disorders,^14–16^ emphasizing its potential role in exacerbating disease risk and prevalence.

More recent inquiry has explored how stress and the social environment can get “into the belly” and affect the diversity, abundance, and function of the gut microbiome^17,18^, the system microorganisms living within the digestive tract. The gut microbiome is recognized as a vital player in the proper functioning of host physiology through the production of neurotransmitters, bile acids, and short-chain fatty acids^19,20^. The “gut-brain axis” is defined as the bidirectional communication between the central and enteric nervous systems, linking emotional and cognitive processes of the brain with peripheral intestinal functioning^21,22^. In interventional research with animals and humans (maternal stress, military training, sleep deprivation, and examination stress), induced stress led to changes in stress response hormones, inflammatory cytokine production, intestinal permeability, gastric motility, and behaviors including anxiety and aggression^22,23^. Some of these results have also been observed in infants following microbiota dysbiosis of the mother during pregnancy^24^. Research is emerging linking subjective measures of stress and induced stress models, such as Cohen’s perceived stress score (PSS), school examinations, circadian disruption, and social defeat models in animals, with changes in gut microbiota diversity and abundance and gastrointestinal disorders^23^. Additionally, research observing associations between gut microbiota and cognitive functioning (emotions, memory, anxiety and depressive feelings) has involved interventions including probiotics, vitamin D, dairy products, and fiber (inulin)^25^. The experience of discrimination in association with metrics of the gut microbiome has not been reported to date, but is paramount to health disparities research and future interventional strategies^18,26^.

The association between life stress and gut microbiota may be explained by physiological and behavioral responses. The experience of stress can have an immediate effect on physiology by activating the stress response system, resulting in increased production of cortisol and inflammatory cytokines^4^. Chronic, on-going stress impairs normal HPA-axis response and immune function, and has been associated with cardiometabolic and gastrointestinal disorders^27,28^. Limited research has examined whether chronic cortisol level, an objective measure of stress, mediates associations between subjective stressors and the gut microbiota. Second, diet is recognized as a major modifiable factor in altering microbiota diversity, abundance, and function^29^. Habitual dietary patterns are associated with microbial clusters, mucosal protection, and anti/pro inflammatory features^30–32^. The intake of micro and macronutrients has also been associated with various gut microbiota taxa, with dietary fiber often being a strong contributor to maintaining bacterial diversity^33,34^. Diet quality has also been found to be highly correlated with various types of stressors and disease mortality^27,35^. This has led some to observe the mediating and moderating role of diet quality in stress-disease processes,^36,37^ but these mediating and moderating inquiries have not yet included the gut microbiome. We propose that a diet score developed to assess the dietary inflammatory potential (dietary inflammation score, DIS) may mediate the association between the experience of discrimination, and other stressors and disease-related gut microbiota. We also recognize that individual nutrients may have specific effects on the gut microbiota; thus we are interested in exploring other potential dietary mediators including the ratio of caloric intake over estimated energy expenditure (cal:EER), healthy eating index (HEI), fiber, sugar, and other dietary variables. This research will add to the limited data related to subjective measures of stress, including the experience of discrimination, perceived stress, and depression, and various dietary variables and the gut microbiome of metabolically healthy young adult women. This study aims to explore the gut microbiota profile of generally healthy young women in relation to diet and stress as well as extend upon the interest in uncovering gut microbiota differences by race. We also aim to explore the potential mediating role of diet and cortisol in the association between reported stress, alpha diversity, and specific stress-related gut microbiota. First, we hypothesize there will be differences in gut genera, dietary, and stress variables between races. Second, we aim to explore associations between stressors and gut microbiota diversity and genus abundance. We hypothesize that stressors will be negatively associated with gut microbiota diversity, positively associated with potential pathogenic bacteria, and negatively associated with healthful bacteria identified through previous research. Third, we aim to uncover associations between diet scores (DIS and HEI), individual dietary variables, and gut microbiota diversity and genus abundance. Observance of similar correlations will lead us to explore the stress-gut relationship through mediation analyses. Last, we hypothesize that cortisol levels (physiological mediator) or diet (nutritional mediator) will mediate the associations found between stressors and gut microbiota diversity or abundance. We are primarily interested in recent (RED) and lifetime experience of discrimination (LED) as the stressors, DIS as the dietary mediator, and hair cortisol (HC) as the physiological mediator; thus all other stress and dietary variables included in the analyses are exploratory. The rationale for this study involves the use of stress measures (discrimination) and a dietary index (DIS) that has not been covered in this area of research, as well as the observance of gut microbiota data of young, generally healthy women, whereas the majority of research is among those with cardiometabolic disease or dysfunction (diabetes). Second, because diet is such a strong moderator of the gut microbiota, we are interested in the independent/dependent association of subjective stress with gut microbiota, and if dietary intake or cortisol levels explain the association between the two. Stress and changes in mood or emotion so often leads to reduced engagement in health promoting behaviors, including low quality diet. To our knowledge and review, this question of dietary mediation has not been observed in stress-gut research.

## Materials and Methods

### Study Participants

Sixty-two African America (AA) and European American (EA) females were recruited from Birmingham, Alabama between August 2014 and April 2016. The same flyer was distributed on all marketing platforms (the University of Alabama at Birmingham [UAB] website, word of mouth, and social media [Facebook]) and included messaging with interest in studying life stress, diet quality, and health status. A screening questionnaire was used to determine eligibility. Participants were eligible if they were under the age of 45 and had a body mass index (BMI) between 18.5 and 45 kg/m^2^. Participants were excluded if they had any medical diagnosis or medication known to affect body composition or metabolism (e.g., diuretics, beta-blockers, calcium channel blockers, angiotensin-converting enzyme inhibitors, angiotensin II receptor blockers, and other hypertension drugs). Participants were also excluded if they were taking monophasic or biphasic oral contraceptive (birth control) or reported an exercise frequency of greater than 2 hours per week, as these types of oral contraceptives^38^ and exercise^39^ alter hormone production, including cortisol, and would confound study results. Triphasic contraceptives are designed to mimic the natural rise and fall of estrogen and progesterone during a menstrual cycle.

### Protocol

Data for this observational study were collected during two visits. Demographics, food frequency questionnaire (FFQ), discrimination and perceived stress questionnaires, and hair and saliva samples were collected at the first visit. Stool samples were collected at home and returned at the second visit, within a week of the first visit. If participants were taking triphasic oral contraceptive, they were scheduled for their visits during their luteal phase, when taking their placebo dose. Luteal phase was determined by patient report. All sample collections and analyses were conducted in the Core Laboratory of the UAB Center for Clinical and Translational Science (CCTS) Clinical Research Unit (CRU), the UAB Diabetes Research Center’s Bio Analytical Redox Biology (BARB) Core, and the UAB Nutrition Obesity Research Center. Recruitment concluded once 31 participants from each race were reached, to ensure no overrepresentation or bias.

### Demographics

Demographics obtained from participants included age, race, income, marital status, and education level. Participants ages ranged from 18 to 45 years, which has been defined as young adult. Income levels ranged from $0 to greater than $100,000, by increments of $10,000. Participants reported marital status as married, never married, separated, divorced, or widowed. Education levels included partial high school, high school graduate, partial college, college graduate, and graduate professional training.

### Cortisol

Salivary cortisol (SC) and hair cortisol (HC) were assessed as acute and chronic objective measures of stress, respectively. Methodology for SC and HC can be found in previously published works ^40^ and from original sources^41–44^. Briefly, participants provided 5ml of saliva in a sterile collection tube over a period of 10 to 30 minutes and at least 60 minutes following their last meal. Hair samples of approximately 6mm in diameter and 3 cm in length were cut from the vertex posterior, as close to the scalp as possible, since hair growth is ~ 1cm/month.

### Gut Microbiome

In the comfort of their homes, participants used testing kits by Zymo Research to obtain fecal samples for isolation of microbial genomic DNA. Participants were instructed to collect an early morning sample or their first bowel movement of the day. Polymerase chain reaction was used on the prepared DNA samples with unique bar-coded primers to amplify the V4 hypervariable region of the 16S ribosomal DNA gene to create an amplicon library from individual samples. Base paired-end reads were sequenced using Illumina MiSeq. Analysis of the sequence data utilized the QIIME-based pipeline21^45^ to produce sample alpha diversity (amplicon sequence variant, ASV) tables. Analysis included quality control, merging of paired-end reads, ASV grouping, and taxonomic assignment. ASVs with average abundance > 0.005% were further processed and grouped by taxonomy. Each sample had at least 20,350 sequences per sample. Alpha diversity, measured by ASV count, and genus abundance were used in analyses for this manuscript.

### Stress Questionnaires

#### Recent and Lifetime Experiences of Discrimination

Details of the discrimination scale developed by Shariff Marco et al is described in previous studies^40,46,47^. The first section of the questionnaire which assesses recent experience of discrimination (RED; within the last year) and lifetime experience of discrimination (LED), was used in this secondary analysis. Briefly, participants reported whether they had experienced examples of unfair treatment, what they felt was attributed to the unfair treatment, and how stressful they perceived these experiences. Recent discrimination sections 1, 2, and 3 include 8, 6, and 1 questions for each section, respectively. Lifetime discrimination sections 1, 2, and 3 include 5, 6, and 1 questions for each section, respectively. Section 1 questions score from 0 to 3 for frequency, section 2 questions are scored by 0 or 1 (No/Yes) for attribution, and section 3 questions score from 0 to 3 for severity. Total scores for RED and LED range from 0 to 33 and 0 to 24, respectively.

#### Perceived Stress Score (PSS)

Cohen’s PSS is composed of 10 questions assessing the experience or appraisal of stress over the last 30 days. An example question asks, *In the last month, how often have you been angered because of things that happened that were outside of your control?* with responses ranging from 0 (*never*) to 4 (*very often*). Reverse scoring is applied to questions 4, 5, 7, and 8 as they were worded in a positive direction and a response such as *very often* would indicate low perceived stress. Scores ranging from 0 to 13 indicate low stress, 14 to 26 indicate moderate stress, and 27 to 40 indicate high stress. In ongoing observational research from years 1983, 2006, and 2009, higher perceived stress scores remain more prevalent among women, minorities, and younger age groups^48^.

#### Patient Health Questionnaire- 8 (PHQ-8)

The eight-item patient health questionnaire has been validated and established as a depression diagnostic tool, measuring the severity of depression disorders^49^. Scores range from 0 to 24 with scores of 0 to 4, 5 to 9, and 10 or greater indicating none-minimal, mild, or moderate/current depression, respectively. Questions assess feelings of interest or pleasure, hopelessness, sleep hygiene, energy, appetite, self-worth, concentration, and communication difficulties over the past 2 weeks, with responses ranging from 0 (*not at all*) to 3 (*nearly every day*). A depression score of 10 or greater, or current depression, has been found to be prevalent among women, but surprisingly, more prevalent among those who are married, employed, college educated, and white race.

#### Food Frequency Questionnaire (FFQ) and Dietary Inflammation Score (DIS)

Dietary intakes were assessed with a validated, self-administered graphical FFQ through VioScreen^50^, which utilizes the food and nutrient information from the Nutrition Coordinating Center (NCC) Food and Nutrient Database. Data from the FFQ were used to calculate a DIS for each participant. The DIS was developed by grouping FFQ foods into 19 food groups, *a priori*, based on biological plausibility and prior literature ^51^. Using multivariable linear regression, the authors determined each DIS food groups weight based on its association with an inflammation biomarker score (hs-CRP, IL-6, IL-8, IL-10). Briefly, scores were calculated by 1) grouping foods of the FFQ by the amount (grams) into DIS food groups, 2) disaggregating mixed dishes into DIS food groups, 3) calculating the supplement score (if data are available), 4) standardizing each food group, 5) multiplying each DIS component by weight, and 6) summing weight values to equal DIS score (more negative values indicate a more anti-inflammatory score). Vitamin and mineral supplementation was not included in the calculation of DIS in our cohort due to inadequate questioning of supplementation. Refer to the supplemental file of the DIS validation study for a step-by-step process in calculating the DIS ^51^. Following protocol established by Byrd et al, participant data were excluded from analyses if the FFQ estimated implausible caloric intake (< 500 and > 4500 kilocalories). Dietary variables of interest gathered from Vioscreen FFQ include: HEI, healthy eating index; cal:EER, the ratio of estimated daily caloric intake to estimated energy requirements (EER); pcarb, percentage carbohydrate intake; pfat, percentage fat intake; pprot, percentage protein intake; bcar, beta carotene (mcg); acar, alpha carotene (mcg); vitc, vitamin c (mg); fiber (g); vitdiu, vitamin D (IU); viteiu, vitamin E (IU); n-3, omega 3; n-6, omega 6; 6:3 ratio, the ratio of omega 6 to omega 3; mfa, monounsaturated fat; pfa, polyunsaturated fat; sfa, saturated fat; tfa, trans fats; sugar, added sugar intake (tsp); fruit, servings of fruit; veg, servings of vegetables; sweet, servings of sweets; and FF, servings of fast food.

### Statistical Analyses

Descriptive statistics (mean, standard deviation [SD], and frequencies) were calculated to summarize demographic characteristics of the cohort. ANOVA was used to determine differences in gut microbiota measures, RED score, dietary variables, DIS by race. Parametric and non-parametric p-values were reported due to violation of the assumptions of homogeneity of variances and normal distribution. Spearman correlation was used to first determine the correlations among stress variables, gut microbiota diversity and the abundance of the top 25 genera of this cohort. Spearman correlation was also used to assess the correlation of various dietary variables and gut microbiota diversity and the abundance of the top 25 genera of this cohort. Mediation analyses were then run to assess the potential mediating role of dietary variables or HC in the significant correlations found between life stressors and gut diversity and genus abundance. Independent, or predictor variables, included RED, LED, and PSS. Dependent, or outcome variables, included ASV count, Alistipes abundance, and Blautia abundance. Mediator variables included HC, DIS, HEI, calories to EER ratio, fiber, and added sugar. Significance was set at α = 0.05 for all statistical analyses. All analyses were performed with SAS statistical software (version 9.4, 2002–2012 by SAS Institute Inc., Cary, NC).

## Results

### Participant Characteristics

Table 1 describes study participants residing in the greater Birmingham area. Women were between the ages of 18 and 45 years with an average of 29 years, and the majority reported partial college education or higher, annual income of greater than $20,000, and never married (Table 1). These characteristics did not significantly differ by race. In Table 2, gut microbiota, dietary, and stress variables are reported by race. ASV count and abundance of genera *Blautia* were lower among AA vs EA women. HEI scores, intakes of fiber, alpha and beta carotene, vitamin E, fruit servings and vegetable servings were lower among AA vs EA, and DIS, sweet servings, and fried food servings were higher among AA vs EA. HC levels were significantly higher in AA vs EA, and AA had significantly higher scores for lifetime discrimination (LED) and PSS compared to EA.

### Correlations between Life Stress Variables and Top 25 Gut Microbiota Genera (Heatmap)

**Figure 1:**
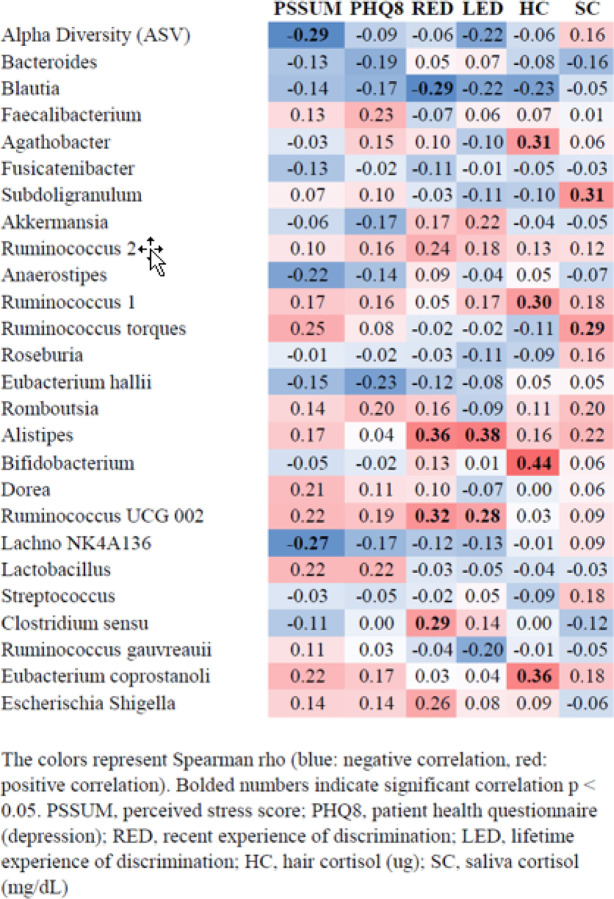
Simple Correlations between Stress Variables and Top 25 Gut Microbiota Genus

Life stress variables were found to be positively and negatively correlated with many of the top 25 genera (Fig. 1). Significant correlations were found between total RED and *Alistipes* (r_s_ 0.36; p = 0.007), *Clostridium sensu* (r_s_ 0.29; p = 0.029), *Blautia* (r_s_ −0.29; p = 0.030), and *Ruminococcus UCG 002* (r_s_ 0.32; p = 0.017). Significant correlations were found between LED and *Alistipes* (r_s_ 0.35; p = 0.004) and *Ruminococcus UCG 002 (*r_s_ 0.28; p = 0.041). Significant correlations were found between PSS and ASV count (r_s_ −0.29; p = 0.029) and *Lachnospiraceae NK4A136 group* (r_s_ −0.27; p = 0.047). No significant correlations were found between PHQ-8 and ASV count or the top 25 genera. Significant correlations were found between HC (ug) and *Agathobacter* (r_s_ 0.31; p = 0.023), *Ruminococcus 1* (r_s_ 0.30; p = 0.027), *Bifidobacterium* (r_s_ 0.44; p = 0.001), and *Eubacterium coprostanoli* (r_s_ 0.36; p = 0.007). Last, significant correlations were found between SC (mg/dL) and *Subdoligranulum* (r_s_ 0.31; p = 0.021), and *Ruminococcus torques* (r_s_ 0.29; p = 0.032).

### Correlations between Dietary Variables and Top 25 Gut Microbiota Genera (Heatmap)

Various dietary variables were correlated with many of the top 25 genera (Figs. 2.1 and 2.2). ASV count was only significantly negatively correlated with trans fat intake (r_s_ −0.29; p = 0.033). *Bacteroides* abundance was significantly negatively correlated with omega 3 intake (r_s_ −0.28; p = 0.039). *Faecalibacterium* abundance was significantly positively correlated with vitamin E intake (r_s_ 0.28; p = 0.036) and monounsaturated fat intake (r_s_ 0.27; p = 0.047). *Fusicatenibacter* abundance was significantly positively correlated with percent protein (r_s_ 0.269; p = 0.047), beta carotene (r_s_ 0.27; p = 0.050), vitamin D (r_s_ 0.54; p < 0.0001), and omega 3 (r_s_ 0.30; p = 0.028). *Akkermansia* abundance was negatively correlated with percent fat intake (r_s_ −0.27; p = 0.047), omega 3 (r_s_ −0.36; p = 0.007), omega 6 (r_s_ −0.33; p = 0.015), mfa (r_s_ −0.30; p = 0.025), pfu (r_s_ −0.34; p = 0.012), and sfa (r_s_ −0.31; p = 0.023). *Anaerostipes* was significantly positively correlated with alpha carotene (r_s_ 0.29; p = 0.038), vitamin C (r_s_ 0.40; p = 0.004), fiber (r_s_ 0.31; p = 0.025), and fruit servings (r_s_ 0.28; p = 0.042). *Ruminococcus torques* was significantly negatively correlated with beta carotene (r_s_ −0.28; p = 0.044) and vegetable servings (r_s_ −0.31; p = 0.022). *Alistipes* was significantly negatively correlated with vegetable servings (r_s_ −0.39; p = 0.004). *Bifidobacterium* was significantly negatively correlated with fruit servings (r_s_ −0.27; p = 0.047) and significantly positively correlated with fast food servings (r_s_ 0.34; p = 0.014). *Dorea* was significantly positively correlated with DIS (r_s_ 0.30; p = 0.031). *Lachnospiraceae NK4A136* group was significantly positively correlated with beta carotene (r_s_ 0.33; p = 0.018), alpha carotene (r_s_ 0.44; p = 0.001), vitamin C (r_s_ 0.47, p 0.000), and fiber (r_s_ 0.37, p = 0.006), and negatively correlated with tfa (r_s_ −0.29; p = 0.032). *Clostridium sensu* was significantly positively correlated with fast food servings (r_s_ 0.29; p = 0.033). Last, *Eubacterium coprostanoli* was significantly negatively correlated with fiber (r_s_ −0.29; p = 0.034).

### Mediating Role of Diet or Cortisol in the Association between Life Stress and Gut Microbiota Diversity and Abundance

**Figure 3:**
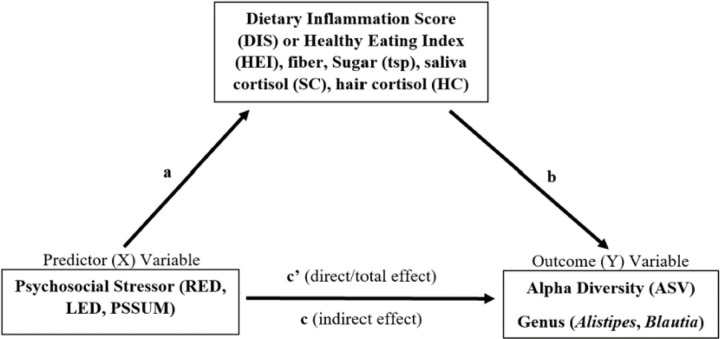
Mediation Concept: The Mediating Role of Dietary and Stress Variables in the Association between Psychosocial Stress and *Alistipes* Abundance, *Blautia*Abundance and Alpha Diversity

There was no mediating effect of dietary variables or cortisol variables in the associations between subjective life stress variables (PSS, RED, LED) and ASV count, *Alistipes* or *Blautia* abundance (Fig. 3, Table 3). We tested the significance of this indirect effect using bootstrapping procedures. Unstandardized indirect effects were computed for each of 5,000 bootstrapped samples, and the 95% confidence interval was computed by determining the indirect effects at the 2.5th and 97.5th percentiles. RED and LED were not significantly associated with any mediator variables. Perceived stress was a better stress predictor of dietary intake (HEI, p = 0.000; DIS, p = 0.001; fiber, p = 0.035), but did not significantly associate with ASV count.

## Discussion

Our results of these analyses reveal interesting findings of the gut microbiota in relation to race, psychosocial stress, and dietary intake. First, ASV count and *Blautia* abundance were significantly lower among AA vs EA. Racial differences of the gut microbiota have been reported in previous yet limited research^26,52,53^, but our findings regarding differences in *Blautia* abundance are new. *Blautia* was recently found to be inversely associated with visceral fat accumulation^54^ and children with obesity and diabetes. A recent literature review of *Blautia* discusses it’s use as a potential beneficial probiotic as it has been found to be involved in flavonoid conversion, free radical scavenging, bacteriocin production thus inhibition of pathogenic bacteria colonization, and maintenance of environmental balance through upregulating T regulatory cells and short-chain fatty acid production^55^.

Several dietary variables were significantly different between AA and EA women. AA women had significantly higher DIS and lower HEI scores, which coincides with higher carbohydrate percentage, fried food servings, and sweet servings, and significantly lower intakes of fiber, beta and alpha carotene, vitamin E, fruit servings, and vegetable servings. Regarding stress variables, AA women also had significantly higher HC levels, and higher reports of lifetime discrimination (LED), and perceived stress (PSS).

Of the top 25 genera of our cohort, *Bacteroides* and *Ruminococcus 1*, *Ruminococcus 2*, and *Ruminococcus torques* were among the most abundant genera and *Bifidobacterium*, *Lachnospiraceae*, and *Lactobacillus* were among the least abundant genera, which are similar gut microbiota characteristics of those following a western, animal-based dietary pattern, high-sugar/high-fat diet, and of individuals who have undergone antibiotic treatments^56,57^. *Blautia* and *Faecalibacterium*, however, were the second and third most abundant genera of the cohort, genera that are reduced in individuals with cardiometabolic risk and disorders and increased with plant intake, respectively. Overall, it appears the cohort has a mixed abundance of bacteria important in various nutrient metabolism and host health.

Next, we observed some significant correlations between stress, dietary variables and gut microbiota diversity and genus abundance. Stress variables were not consistently or similarly correlated with gut diversity or genus abundance. PSS was negatively correlated with ASV count and *Lachnospiraceae NK4A136* group. Depression did not correlate with any metrics. RED scores were correlated with more genera than any other stressor, with stronger correlations found with *Blautia* (negative), *Alistipes* (positive), *Ruminococcus UCG 002* (positive), and *Clostridium sensu* (positive). LED scores were correlated with *Alistipes* (positive) and *Ruminococcus UCG 002* (positive). Hair cortisol was positively correlated with *Agathobacter, Ruminococcus 1, Bifidobacterium*, and *Eubacterium coprostanoli.* Last, salivary cortisol was positively correlated with *Subdoligranulum* and *Ruminococcus torques*.

Regarding diet quality and gut microbiota diversity and abundance, trans fat intake was negatively correlated to ASV, which is similar to recent studies observing dietary fat intake and reduced alpha diversity^58–60^. Adversely, high-glucose and high-fructose diets administered in mice have shown to have this effect on the alpha diversity and increases in proteobacteria phylum, one of the best sources of lipopolysaccharide (LPS), which trigger activation of the innate immune system and inflammatory conditions^61^. Variables thought to effect gut bacteria diversity were not correlated with ASV including high fiber food groups (fruits, vegetables, fiber), cal:EER ratio (energy balance), and fried food servings. Fruit servings, alpha carotene, vitamin C, and fiber were all positively correlated with the genus *Anaerostipes*, a genus that has been found to increase in abundance following the consumption of prebiotic inulin, and improve reports of constipation and stool consistency^62^, possibly through its role in producing butyrate from lactate, contributing to colonic and GI health^63^. Alpha and beta carotene, vitamin C, and fiber were also positively correlated with *Lachnospiraceae NK4A136*, however, this family of bacteria has been found to be controversial in its role in health and disease^64^. *Lachnospiraceae NK4A136* was recently found to be restored after completion of a high-fat diet protocol that induced dysbiosis in mice^65^, was found to be diminished in a small pilot study including individuals with dementia^66^, and was among other short-chain fatty acid producing bacteria that were increased following an inflammation-reducing prebiotic trial in mice^67,68^. More recently, a 4-week tannin supplementation trial in humans was found to increase the abundance of healthy gut bacteria, including *Lachnospiraceae NK4A136*, and increase short chain fatty acid production^69^.

Variables of fat intake were inversely correlated with *Akkermansia*, which is consistent with studies reporting bodyweight and high-fat diet (HFD) in children and pregnant women^70–72^. *Akkermansia* supplementation in mice with high-fat induced obesity led to beneficial effects on weight, blood glucose control, and memory decay^70^. Physiologically, *Akkermansia* administration has been shown to improve insulin sensitivity, attenuate adaptive changes related to caloric intake following cold exposure (negative energy balance), increase fat browning, induce anti-inflammatory effects through Treg cell induction in adipose tissue, and provide protective effect against atherosclerosis^72^. Interestingly, HFD has been shown to decrease *Akkermansia* abundance while fish oil consumption has been shown to increase *Akkermansia*^72,73^. Vegetable servings were inversely correlated with *Alistipes*, another controversial genus^74^ that has been shown to increase in individuals following a calorie restricted high-fat diet that induced weight loss.

Some of our correlational findings between dietary variables and gut microbiota genera were unexpected. For example, fiber was inversely correlated with *Eubacterium coprostanoli*, a genus that has been found aid in the conversion of cholesterol to coprostanol, which is important in cholesterol excretion^75^. Next, *Lactobacillus* was not correlated any dietary variables. Because strains of *Lactobacillus* are often one of the main sources of bacteria in probiotics, inquiry into the associations and effects of different strains on human health is ongoing and somewhat controversial as obese individuals were found to have less *L. caseae* and *L. plantarum*, and greater abundance of *L. reuteri*^76^. Last, the inflammatory score of one’s dietary pattern, captured by DIS, did not correlate with specific gut genera or diversity, unlike findings from a similar study by Zheng et al^77^ who found differentially abundant species between dietary inflammatory index (DII) tertiles, and research observing adherence to Mediterranean diet and beneficial changes in gut microbiota abundance^78^. This may be due to potential significant compositional differences at both ends of the DIS spectrum (animal vs. plant-dominant patterns), and thus the most diverse microbiota may be characterized with a more neutral or balanced DIS.

Finally, variables we hypothesized to mediate the association between subjective stress and gut microbiota parameters did not hold true. In the current study, AA women reported higher perceived stress and lifetime discrimination, greater intakes of sweet and fried food servings, lower diet quality (increased DIS and reduced HEI scores), increased hair cortisol, and reduced ASV count. We hypothesized that a worse dietary intake or elevated cortisol in response to stress may be a way that AA have reduced alpha diversity, increased *Alistipes*, and reduced *Blautia* abundance. Although diet did not mediate the association between PSSUM and *ASV count*, the simple regressions between PSSUM and dietary variables (fiber, DIS, HEI) were significant (Table 3). Additionally, RED and LED were independently significantly associated with *Alistipes* and *Blautia* abundance. These separate associations are significant and reveal the independent associations between stress, diet quality, and specific gut bacteria abundance. Previous research has similarly explored associations between added sugar intake, the abundance of various pathogenic gut bacteria, and cognitive function. Added sugar intake was found to increase inflammatory-related bacteria such as *Proteobacteria*, reduce beneficial bacteria, *Bacteroidetes*^72^ and *Lachnobacterium*^73^, and increase species of *Parabacteroides*, which were found to impair memory performance^74^. The alterations in these bacteria abundances can lead to lipopolysaccharide-induced inflammation and impaired gut integrity through modified tight junctions and increased intestinal permeability.

Still, subjective stress questionnaires may not be the best predictor of dietary, physiologic, or gut microbiota metrics because of the nature of the variable accounting for past and cumulative experience, not current or acute response. Additionally, RED, LED, and other discrimination questionnaires do not capture one’s resilience or emotional response to these experiences. Induced stress interventions, such as the Trier social stress test^79^, and observation of immediate food choice, stress response, and gut microbiota changes will better capture these relationships and mediations. Further research in this area is important to explore effective protocols in reducing the burden of stress.

This research is not without limitations. First, the cross-sectional, observational design of this study cannot describe causal relationships. Additionally, the sample size and metabolically healthy state of our cohort may limit the strength of our findings. Prospective studies observing the gut microbiota across the young adult period would be beneficial in learning about the resilience of one’s gut microbiome and any significant changes that may be associated with declining or stable health, chronic experiences of stress, and diet quality. Future research should continue to observe the relationships among stress, diet, and the gut microbiome in early adolescence and young adulthood and conduct interventions involving mental health treatment and lifestyle modification, with hopes to halt or reverse concerning trends in obesity, prehypertension and hypertension, and pre-diabetes and diabetes rates of this population^14, 80–82^.

## Conclusion

In conclusion, we observed several disparities between races in diet and stress but few differences in microbiota. We also found correlations between the top 25 gut microbiota genera and diet quality (animal, plant, fat, fiber, etc) to be mixed. To date, it appears that greater microbial diversity is indicative of resilience and good health^83^, and reduced diversity is associated with autoimmune disorders and cardiometabolic disease^20^. There was not partial or full mediation by dietary or cortisol variables in the associations between subjective stress and alpha diversity or gut bacteria abundance.

The associations between perceived stress and fiber, DIS, and HEI is important to note as young adults, especially those between the ages of 18 and 25, were shown to have experienced the greatest increase in symptoms consistent with major depression, suicidal thoughts, and serious psychological distress over the last decade (2008–2017). The prevalence of mental illness symptoms is cause for concern as individuals are less likely to engage in health-promoting behaviors. Choosing low-nutrient, high-calorie, sugary and fatty foods as a response to stress may alter the health and diversity of a young individuals gut microbiome, potentially leading to changes in metabolic health.

## Figures and Tables

**Figure 2:**
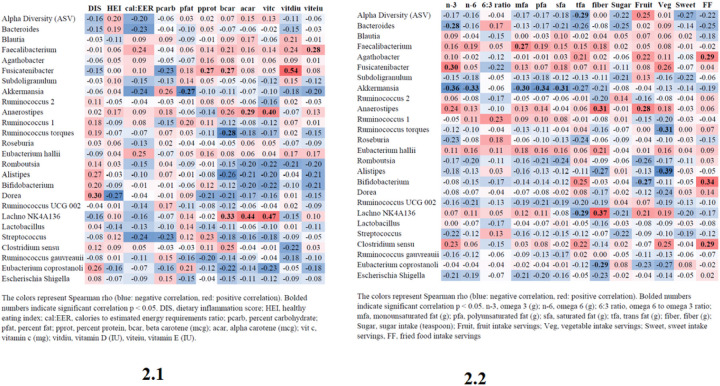
2.1 Simple Correlations between Dietary Variables and Top 25 Gut Microbiota Genus 2.2 Simple Correlations between Dietary Variables and Top 25 Gut Microbiota Genus

**Table 1:** Descriptive characteristics of African American and European American women from Birmingham, AL

Variables	African Americans (N = 29)	European Americans (N = 31)
	**Percentage (n)**	

**Marital status**		
**Divorce**	**17.24% (n = 5)**	**6.45% (n = 2)**
**Never married**	**62.07% (n = 18)**	**67.74% (n = 21)**
**Married**	**20.69% (n = 6)**	**25.81% (n = 8)**

**Income (annual)**		
$0 (no income)	6.90% (n = 2)	3.23% (n = 1)
<$20,000	27.58% (n = 8)	16.13 (n = 5)
≥$20,000	65.54% (n = 19)	80.65 (n = 25)

**Education**		
Partial High School	3.23% (n = 1)	NA
High School Graduate	12.90% (n = 4)	6.45% (n = 2)
Partial College	38.71% (n = 10)	32.26% (n = 10)
Standard College Graduate	32.26% (n = 10)	25.81% (n = 8)
Graduate Professional Training	12.90% (n = 4)	35.48% (n = 11)

	**Mean ± SD**	

**Age (years)**	29.07 ± 7.83	28.83 ± 7.28

**Table 2:** Gut Microbiome, Diet, and Psychosocial Stress Variables by Race

Top 25 Genera	AA (N = 29) Mean ± SD	EA (N = 31) Mean ± SD	p-value	AA Median	EA Median	p-value
Alpha Diversity (ASV)	95.73 ± 27.52	113.48 ± 32.29	**0.032**	98.5	107	0.063
Bacteroides	0.233 ± 0.143	0.239 ± 0.128	0.877	0.201	0.238	0.736
Blautia	0.118 ± 0.065	0.162 ± 0.081	**0.030**	0.110	0.158	**0.035**
Faecalibacterium	0.095 ± 0.068	0.090 ± 0.061	0.808	0.091	0.080	0.946
Agathobacter	0.031 ± 0.034	0.038 ± 0.051	0.564	0.022	0.023	0.973
Fusicatenibacter	0.033 ± 0.026	0.030 ± 0.019	0.609	0.027	0.034	0.859
Subdoligranulum	0.026 ± 0.020	0.028 ± 0.023	0.736	0.024	0.028	0.893
Akkermansia	0.019 ± 0.035	0.016 ± 0.034	0.768	0.000	0.002	0.480
Ruminococcus 2	0.014 ± 0.018	0.017 ± 0.018	0.395	0.000	0.015	0.220
Anaerostipes	0.017 ± 0.015	0.018 ± 0.016	0.937	0.015	0.014	0.893
Ruminococcus 1	0.015 ± 0.021	0.017 ± 0.020	0.719	0.005	0.009	0.665
Ruminococcus_torques	0.016 ± 0.015	0.016 ± 0.013	0.931	0.010	0.014	0.800
Roseburia	0.015 ± 0.022	0.015 ± 0.012	0.901	0.008	0.014	0.206
Eubacterium_hallii	0.013 ± 0.010	0.015 ± 0.009	0.391	0.012	0.012	0.409
Romboustia	0.014 ± 0.017	0.014 ± 0.018	0.960	0.008	0.005	0.912
Alistipes	0.015 ± 0.014	0.010 ± 0.008	0.111	0.013	0.008	0.288
Bifidobacterium	0.020 ± 0.033	0.006 ± 0.008	**0.037**	0.003	0.002	0.402
Dorea	0.011 ± 0.009	0.010 ± 0.006	0.441	0.009	0.008	0.906
Rumin 002	0.012 ± 0.015	0.008 ± 0.009	0.189	0.006	0.005	0.466
Lachno NK4A136	0.005 ± 0.006	0.010 ± 0.014	0.088	0.003	0.004	0.261
Lactobacillus	0.004 ± 0.012	0.001 ± 0.003	0.163	0.000	0.000	0.172
Streptococcus	0.009 ± 0.013	0.007 ± 0.011	0.501	0.004	0.003	0.417
Clostridium_sensu	0.009 ± 0.017	0.006 ± 0.011	0.399	0.003	0.001	0.138
Ruminococcus_gauvreauii	0.006 ± 0.007	0.008 ± 0.010	0.323	0.004	0.006	0.708
Eubacterium coprostanoli	0.007 ± 0.009	0.006 ± 0.007	0.839	0.004	0.004	0.932
Escherischia Shigella	0.007 ± 0.018	0.007 ± 0.017	0.959	0.000	0.001	0.835
Dietary Variables	AA	EA	p-value	AA	EA	p-value
Dietary Inflammation Score (DIS)	0.79 ± 2.27	−0.931 ± 2.55	**0.010**	0.55	−1.32	**0.004**
Healthy Eating Index (HEI) Score	62.3 ± 11	69.3 ± 11.2	**0.021**	59.9	70.8	**0.014**
Cal:EER ratio	0.90 ± 0.37	0.93 ± 0.36	0.750	0.81	0.88	0.575
Percent Carb	52 ± 9	47 ± 10	0.054	48	46	**0.049**
Percent Fat	34 ± 5	37 ± 8	0.082	34	36	0.089
Percent Protein	15 ± 4.5	16 ± 3	0.156	15	17	0.095
Fiber (g)	19.6 ± 9.1	28.5 ± 16.4	**0.015**	17.7	26.7	**0.014**
Beta Carotene (mcg)	5401 ± 8308	9468 ± 10838	0.119	2378	6027	**0.012**
Alpha Carotene (mcg)	456 ± 499	1779 ± 2631	**0.012**	249	700	**0.008**
Vitamin C (mg)	147 ± 115	173 ± 187	0.540	114	136	0.453
Vitamin D (IU)	264 ± 200	259 ± 134	0.914	219	260	0.412
Vitamin E (IU)	16.8 ± 9.2	25 ± 17.4	**0.030**	13.5	21.6	**0.025**
Omega 6:3	8.2 ± 1.9	9.3 ± 2.6	0.086	8.5	8.7	0.236
Added Sugar (tsp)	21.3 ± 17.6	13.6 ± 15.2	0.083	15.5	10.5	0.074
Sweet Servings	3 ± 3	1.5 ± 2.2	**0.031**	1.8	0.66	**0.010**
Fruit Servings	0.88 ± 1.1	1.8 ± 1.5	**0.012**	0.50	2	**0.006**
Vegetable Servings	1.3 ± 1.8	2.1 ± 1.9	0.098	0.50	2	**0.026**
Fried Food Servings	0.50 ± 0.40	0.24 ± 0.27	**0.015**	0.31	0.15	**0.003**
Psychosocial Stress Variables	AA	EA	p-value	AA	EA	p-value
Hair Cortisol (HC)	0.113 ± 0.227	0.035 ± 0.033	0.080	0.043	0.024	**0.008**
Saliva Cortisol (SC)	0.224 ± 0.144	0.173 ± 0.101	0.121	0.182	0.158	0.185
Recent Discrimination(RED)	10.8 ± 5.8	8.4 ± 6.1	0.174	10.5	6.5	0.103
Lifetime Discrimination (LED)	9.5 ± 4	6.7 ± 3.5	**0.014**	9.5	6	**0.011**
Perceived Stress Score (PSS)	20 ± 6.5	15.2 ± 6.8	**0.007**	20	13	**0.008**
Depression (PHQ-8)	7 ± 5.4	6 ± 5.3	0.544	5	5	0.423

* Mean ± SD and p values were determined using ANOVA.

* Median and p values were determined using Kruskal-Wallis test.

P values for unequal variances were determined using Welch’s ANOVA.

**Table 3:** Mediation Analysis: Psychosocial Stress, Diet, and the Gut Microbiota

X	Mediator	Y	Path a		Path b		Path c’		Path c	
			b	p-value	b	p-value	b	p-value	Adj R	p-value
RED	Fiber	*Alistipes*	−0.642	0.103	−0.0001	0.389	0.001	**0.029**	0.093	0.065
RED	DIS	*Alistipes*	0.132	**0.054**	0.001	0.198	0.001	**0.029**	0.116	**0.041**
RED	HEI	*Alistipes*	−0.074	0.803	−0.0000	0.993	0.001	**0.029**	0.074	0.095
RED	SC	*Alistipes*	0.002	0.604	0.028	0.126	0.001	**0.024**	0.134	**0.024**
LED	Fiber	*Alistipes*	−0.391	0.514	−0.000	0.285	0.001	**0.026**	0.104	**0.049**
LED	DIS	*Alistipes*	0.058	0.572	0.001	0.138	0.001	**0.026**	0.130	**0.029**
LED	HEI	*Alistipes*	0.119	0.799	0.000	0.943	0.001	**0.026**	0.076	0.087
LED	HC	*Alistipes*	0.008	0.341	0.011	0.172	0.001	0.133	0.057	0.127
RED	Fiber	*Blautia*	−0.650	0.115	−0.0003	0.570	−0.003	**0.054**	0.059	0.136
RED	DIS	*Blautia*	0.111	0.116	−0.002	0.594	−0.003	**0.054**	0.057	0.139
RED	HEI	*Blautia*	0.057	0.849	−0.0002	0.757	−0.003	**0.054**	0.052	0.152
RED	SC	*Blautia*	0.002	0.450	−0.042	0.593	−0.003	**0.019**	0.101	**0.056**
LED	Fiber	*Blautia*	−0.391	0.514	0.0004	0.608	−0.003	0.215	−0.005	0.410
LED	DIS	*Blautia*	0.058	0.572	−0.003	0.566	−0.003	0.215	−0.003	0.340
LED	HEI	*Blautia*	0.119	0.799	−0.001	0.599	−0.003	0.215	−0.004	0.408
LED	HC	*Blautia*	0.006	0.431	−0.081	0.178	−0.004	0.183	0.041	0.167
LED	Fiber	ASV	−0.276	0.499	0.024	0.927	−1.188	0.110	0.012	0.282
LED	DIS	ASV	0.006	0.933	−1.234	0.375	−1.188	0.110	0.028	0.191
LED	HEI	ASV	0.014	0.967	0.333	0.298	−1.188	0.110	0.034	0.164
LED	HC	ASV	0.005	0.260	−4.260	0.866	−1.426	0.104	0.013	0.265
LED	Sugar	ASV	0.319	0.492	−0.119	0.606	−1.188	0.110	0.017	0.248
PSSUM	Fiber	ASV	−0.571	**0.035**	−0.164	0.595	−1.04	0.077	0.028	0.185
PSSUM	DIS	ASV	0.169	**0.001**	−0.649	0.717	−1.04	0.077	0.025	0.200
PSSUM	HEI	ASV	−0.782	**0.000**	0.284	0.490	−1.04	0.077	0.032	0.168
PSSUM	Sugar	ASV	0.617	0.063	−0.289	0.248	−1.04	0.077	0.048	0.109

## Abbreviations

AA: African American
EA: European American
PSS: perceived stress score
DIS: dietary inflammation score
cal: EER:ratio of caloric intake over estimated energy expenditure
HEI: healthy eating index
RED: recent experience of discrimination
LED: lifetime experience of discrimination
HC: hair cortisol
BMI: body mass index
FFQ: food frequency questionnaire
SC: salivary cortisol
ASV: amplicon sequence variant
PHQ8: patient health questionnaire-8 (depression)
pcarb: percentage carbohydrate intake
pfat: percentage fat intake
pprot: percentage protein intake
bcar: beta carotene (mcg)
acar: alpha carotene (mcg)
vitc: vitamin c (mg)
vitdiu: vitamin D (IU)
viteiu: vitamin E (IU)
n-3: omega 3
n-6: omega 6
6: 3 ratio:the ratio of omega 6 to omega 3
mfa: monounsaturated fat
pfa: polyunsaturated fat
sfa: saturated fat
tfa: trans fats
sugar: added sugar intake (tsp)
fruit: servings of fruit
veg: servings of vegetables
sweet: servings of sweets
FF: servings of fast food
